# Editorial: Does the golgi complex enable oncogenesis?

**DOI:** 10.3389/fcell.2022.1000946

**Published:** 2022-08-30

**Authors:** Antonino Colanzi, Setharaman Parashuraman, Celso A. Reis, Daniel Ungar

**Affiliations:** ^1^ Institute of Experimental Endocrinology and Oncology “G. Salvatore” (IEOS), National Research Council (CNR), Naples, Italy; ^2^ Department of Biotechnology, Bhupat and Jyoti Mehta School of Biosciences, Indian Institute of Technology-Madras, Chennai, India; ^3^ Institute for Research and Innovation in Health—i3S, University of Porto, Porto, Portugal; ^4^ Institute of Molecular Pathology and Immunology of the University of Porto, Porto, Portugal; ^5^ Institute of Biomedical Sciences of Abel Salazar, University of Porto, Porto, Portugal; ^6^ Department of Biology, University of York, York, United Kingdom

**Keywords:** golgi apparatus glycosylation, golgi structure, cancer, mitosis, signaling

The Golgi complex (GC) is a fundamental element of the secretory pathway (Glick and Nakano, 2009). In vertebrate cells, the GC is generally organized in the form of functionally polarized stacks of cisternae, each containing a distinct set of cargo-processing enzymes (Wei and Seemann, 2010). An additional level of organization is present in mammalian cells, where adjacent stacks are connected by membranous tubules (Rambourg and Clermont, 1990) to form a single copy organelle (Cole et al., 1996), called Golgi ribbon, often in close proximity to the centrosome (Wei and Seemann, 2010). The proteins and lipids that are synthesized in the endoplasmic reticulum (ER) are transported to the cis-Golgi through an intermediate compartment (Appenzeller-Herzog and Hauri, 2006). The cargoes are then processed through the GC and sorted at the trans-Golgi network (TGN) for transport to specific plasma membrane domains or organelles (Glick and Nakano, 2009). As it is oft-repeated, the Golgi apparatus (and secretory pathway) is responsible for producing and positioning nearly one-third of the proteome and thus is a primary organizer of the mammalian cell. This is a process in which Golgi-resident glycosyltransferases play a major role by generating protein diversity and affecting many physiological and pathological processes (Pinho and Reis, 2015; Bellis et al., 2022).

The complex structural organization of the Golgi apparatus both at the level of the stack and at the level of the Golgi ribbon has important functional implications. The polarized organization of the Golgi apparatus stack has been shown to regulate the efficient and faithful glycosylation of cargoes (Fisher et al., 2019; Pothukuchi et al., 2019; Pothukuchi et 2021). On the other hand, the ribbon organization has also been demonstrated to increase the efficiency of glycan processing (Puthenveedu et al., 2006) and in addition, also regulates transport and polarized delivery of selected cargo, and signaling events at the Golgi (Makhoul et al., 2018; Kulkarni-Gosavi et al., 2019; Li et al., 2019; Ravichandran et al., 2020), in line with the well-recognized role as an intracellular signaling platform (Luini and Parashuraman, 2016).

All of these activities—cargo transport and sorting, cargo processing, such as glycosylation, and being a platform for signal transduction may have oncogenic effects when defective, and are discussed in this Research Topic of Frontiers in cell and developmental biology. The evidence presented in this issue shows that the role of the GC is not “limited” to its established fundamental role as a central station for processing and trafficking of proteins and lipids. Rather, it is also a central signaling hub regulating key cellular functions—including cell proliferation, cell migration, proteostasis, and apoptosis.

The important contribution of Golgi structure to its function is reflected in the diverse changes in this structure seen in cancer cells and tissues, ranging from fragmentation to honeycomb-like organization (Kellokumpu et al., 2002; Sano et al., 2002). These alterations are thought to be fine-tuned adaptations of the organelle to modulate its functions so as to respond to specific cellular needs. While in many cases the structure-function correlation of the GC is not very clear, the fragmentation of the Golgi ribbon is an exception, where the functional relevance of the altered structure has been well-studied. The Golgi ribbon exists as an interlinked ribbon in interphase cells, and is fragmented at the G2 phase, which acts as a checkpoint to allow cells to enter into mitosis when the organelle is further fragmented into smaller vesicles (Sutterlin et al., 2002; Corda et al., 2012; Ayala and Colanzi, 2022). The fragmented Golgi is then reassembled into a ribbon as the cells enter G0/G1 phase. These transitions in Golgi structure are regulated processes controlled by signaling pathways that regulate cell division and impairment of these structural transitions of Golgi apparatus lead to centrosome and chromosome segregation defects (Mascanzoni et al., 2022). Given the important role of the Golgi apparatus in controlling cell division, it is not surprising that the Golgi apparatus acts as a signal integration hub for both internal and external stressors. Bui et al. discuss how these structural changes in the Golgi affect several functions of the organelle including transport, processing and signal transduction. These contribute to several oncogenic features including uncontrolled cell proliferation, enhanced cell migration, anoikis resistance, and immune evasion. Further, structural transitions of the GC are also a part of the EMT program that contributes to cell migration. While the downstream effects of the Golgi structural changes are important, how these changes are achieved in cancer cells is not well understood. Examination of cancer tissues and cells has shown varied alterations of Golgi structure with the fragmentation of the GC being the most frequently observed phenotype. Zhang et al. discusses how structural and functional aspects of the Golgi apparatus are achieved by the altered expression of Golgi matrix proteins in cancer cells. Recent studies have shown that copy number alteration is an important mechanism by which the expression of these proteins is altered in cancers (Tan et al., 2020; Russo et al., 2022). Sahu et al. present and discuss a novel mosaic cisternal maturation framework that provides a model to understand how the altered expression of Golgi transport machinery including the matrix proteins can lead to impaired or altered glycosylation function, which in turn leads to changes in the oncogenic signaling pathways. Given the importance of Golgi structure in the regulation of oncogenic functions including cell proliferation and cell migration, it is not surprising that Golgi structure is under strict regulation by signaling pathways. An important group of these pathways—autoregulatory signaling systems - regulate the morphology and activity of the secretory pathway. Many of the components of these autoregulatory signaling systems, like Src kinase, have known oncogenic roles. Giudice et al. discuss the implications of this intersection of autoregulatory signaling systems of the secretory pathway with oncogenic signaling pathways. Finally, while not directly linked to its secretory function, the Golgi apparatus can also moonlight as a store of H+ ions. It is well known that the pH of the Golgi apparatus is acidic with the trans-Golgi being more acidic than the cis-Golgi. Galenkamp and Commisso discuss how this property of the Golgi apparatus is utilized by cancer cells that use the Golgi as a “proton sink” to maintain an alkaline cytosolic pH. An alkaline cytosolic pH is characteristic of cancer cells and it is necessary for the optimal functioning of glycolytic enzymes that contribute to the Warburg effect. In sum, this series of reviews highlight the emerging role of GC functions in oncogenesis and suggest how Golgi functions can “enable” cancer development and growth, linking this important cellular organizer to many instances of rogue cell biology.
